# Biofluid‐specific variations in circulating 5′ transfer RNA fragments during ictal and interictal states in experimental temporal lobe epilepsy

**DOI:** 10.1002/epi.70246

**Published:** 2026-04-13

**Authors:** Marie Soukupova, Elena Perez Morrissey, Annunziata Guarino, Pietro Marino, Cristiana Pareo, Nicolò Birtolo, Saad Zaheer, Rachel Stewart, Ina Woods, Felix Rosenow, Hajo Hamer, Péter Körtvélyessy, Shona Pfeiffer, David C. Henshall, Jochen H. M. Prehn, Michele Simonato

**Affiliations:** ^1^ Section of Pharmacology, Department of Neuroscience and Rehabilitation University of Ferrara Ferrara Italy; ^2^ Department of Physiology and Medical Physics Royal College of Surgeons in Ireland University of Medicine and Health Sciences Dublin Ireland; ^3^ FutureNeuro Research Ireland Centre Royal College of Surgeons in Ireland University of Medicine and Health Sciences Dublin Ireland; ^4^ Epilepsy Center Frankfurt Rhine of Goethe University Frankfurt am Main Germany; ^5^ Department of Neurology, Epilepsy Center, Kopfkliniken University of Erlangen‐Nürnberg Erlangen Germany; ^6^ Department of Neurology Charité–Universitätsmedizin Berlin, corporate member of Freie Universität Berlin and Humboldt‐Universität zu Berlin Berlin Germany; ^7^ Division of Neuroscience Istituto di Ricovero e Cura a Carattere Scientifico San Raffaele Scientific Institute Milan Italy

**Keywords:** biomarker, blood, cerebrospinal fluid, treatment‐resistant epilepsy, tRNA fragment

## Abstract

**Objective:**

Circulating small noncoding RNAs represent potential biomarkers of temporal lobe epilepsy (TLE). Recently, two transfer RNA fragments (tRFs), 5′tRF Glu‐CTC and Gly‐GCC, were found to be elevated in plasma samples collected in advance of a seizure in TLE patients, suggesting they may serve as potential wet biomarkers of seizure risk or occurrence. The optimal biofluid source for tRF assay, species conservation, and whether their levels reflect specific ictal or interictal states remain uncertain.

**Methods:**

Here, we analyzed the longitudinal dynamics of 5′tRF Glu‐CTC and Gly‐GCC in both cerebrospinal fluid (CSF) and plasma in the pilocarpine model of TLE in rats. We sampled CSF and plasma across multiple time points during the chronic phase of TLE, while performing continuous electroencephalographic monitoring to correlate 5′tRFs levels with electrophysiological activity.

**Results:**

Levels of both 5′tRFs were significantly higher in plasma but not in CSF in epileptic rats compared to controls. Plasma levels of both did not, however, correlate with seizure frequency. In contrast, 5′tRF Gly‐GCC levels in CSF correlated with interictal spike activity, whereas plasma levels again did not show a dynamic response. Consistent with these findings, we observed higher levels of 5′tRFs in plasma but not in CSF samples from human TLE patients as compared with healthy controls.

**Significance:**

These data suggest that 5′tRFs accumulate in the brain during interictal spike activity and accumulate in the plasma of individuals with epilepsy. 5′tRFs may therefore serve as accessible, diagnostic biomarkers for epilepsy.

**Key points**

- Plasma levels of 5′tRFs Glu‐CTC and Gly‐GCC are elevated in epileptic rats and human TLE patients.
- Levels of these tRFs are higher in plasma than in CSF of epileptic rats and human TLE patients.
- CSF levels of 5′tRF Gly‐GCC correlate with interictal spike activity.
- Increased plasma levels of 5′tRFs Glu‐CTC and Gly‐GCC may represent diagnostic biomarkers of epilepsy.

## 1 INTRODUCTION

Epilepsy is a neurological disorder characterized by recurrent seizures that, globally, affects more than 50 million people.^1^ Seizures are classified as focal seizures that affect one specific area of the brain and generalized seizures that affect both hemispheres.^2^ Mesial temporal lobe epilepsy (TLE) is the most common and treatment‐resistant form of focal epilepsy in adults, characterized by spontaneous recurrent seizures (SRSs) arising from the hippocampus and adjacent structures and interictal epileptiform activity, in particular abnormal electroencephalographic (EEG) patterns marked by interictal spikes (ISs) that occur between the seizures. In animal models of TLE, ISs emerge following status epilepticus (SE) and precede the development of SRSs.^3^, ^4^ Clinically, increased interictal spikes activity (ISA) in TLE patients has been linked to cognitive impairments, particularly in learning and memory.^5^

Clinicians, patients, and their carers would greatly benefit from biomarkers that can be sampled by a simple, minimally invasive method to help diagnosing epilepsy or, even more importantly, to predict when a seizure will occur. Although some progress has been made in identifying potential prodromal biomarkers of seizure imminence,^6^, ^7^ a reliable biomarker for an accurate prediction of seizures has not yet been implemented clinically.^8^, ^9^ Small noncoding RNAs (sncRNAs) can be detected in all biofluids including blood (serum, plasma) and cerebrospinal fluid (CSF) and are among the leading molecules being explored as seizure biomarkers.^9^, ^10^, ^11^ However, it is unknown whether CSF or plasma/serum represent the optimal biological fluid for their detection, how this varies according to the species of sncRNA, and whether the biofluid source reflects events during seizures, ISA, or both. In a recent study, we identified three 5′ transfer RNA (tRNA) fragments (tRFs)—5'tRF Gly‐GCC, 5'tRF Ala‐TGC, and 5'tRF Glu‐CTC—that were significantly elevated in plasma samples from TLE patients collected prior to seizure onset compared to postseizure and healthy control samples.^12^ The tRNA‐derived small RNAs (tsRNAs) are a class of sncRNAs produced through the enzymatic cleavage of tRNAs. 5′tRFs are generated by cleavage of tRNAs within the D‐loop by the ribonuclease Dicer.^13^, ^14^ These fragments exhibit high stability in the bloodstream, making them promising candidates for noninvasive biomarker detection.^15^ It is unknown, however, which RNA signals are carried by which biofluid in terms of reflecting preseizure, between seizure (interictal), or postseizure states.^16^ This raises the prospect of assaying multiple sources to implement circulating biomarkers for epilepsy in the clinic or at‐home setting. The degree to which these tRF signatures are conserved in model species (e.g., rodents with epilepsy) is also unknown. Additionally, longitudinal studies analyzing 5′tRF levels in plasma or CSF in patients in relation to seizures and ISA have not been conducted and are ethically and logistically challenging in humans.

To address these gaps, the present study explored the longitudinal dynamics of two selected tRFs, 5'tRF Gly‐GCC and 5'tRF Glu‐CTC, in both CSF and plasma, by sampling across multiple time points during the chronic phase after development of epilepsy induced by pilocarpine in rats, an established animal model of TLE.^17^, ^18^ EEG recordings and IS activity were used to determine whether tRF fluctuations in peripheral blood and CSF changes contained similar or different information on seizure states. Clinical validity was assessed using a set of plasma and CSF samples from TLE patients. Our findings reveal that different biofluid compartments display seizure state‐specific information, providing insight to the origin of the signals and their practical use as sources of epilepsy biomarkers.

## 2 MATERIALS AND METHODS

A detailed description of the methods, procedural details, and experimental analysis related to the animal study are presented in the Supplementary Information. All procedures were approved by the University of Ferrara institutional animal care and use committee and by the Italian Ministry of Health (authorization: D.M. 603/2022‐PR).

### 2.1 CSF and plasma collection from rats

CSF and blood were longitudinally sampled during the late chronic phase via the cisterna magna (CSF) and tail vein (blood) at 53, 56, 59, 62, and 65 days after SE. CSF and plasma sampling was performed as described previously.^19^ Rats were briefly anesthetized with isoflurane (5% induction, 1%–2% maintenance). First, ~100 μL of CSF was drawn from the cisterna magna using a 23‐G butterfly needle, transferred into a .2‐mL Eppendorf tube, and frozen at −80°C. Immediately after CSF withdrawal, .5 mL of blood was drawn from the lateral tail vein using a 21‐G butterfly needle and transferred into BD Microtainer K2 EDTA tubes (BD Biosciences). Plasma was isolated by centrifuging blood samples at 1300 × *g* for 10 min at 4°C and stored at −80°C.

### 2.2 RNA extraction

RNA was extracted from CSF and plasma using the miRNeasy Serum/Plasma Advanced Kit (Qiagen, catalog no. 217204) with the addition of the RNA spike‐in *Cel‐miR‐39‐3p* (Qiagen, catalog no. 339390) as per the manufacture's instruction. The final elution of RNA was performed using 20 μL of DNase/RNase‐free water, and samples were stored at −80°C.

### 2.3 TaqMan real‐time quantitative polymerase chain reaction of samples

Quantitative polymerase chain reaction (qPCR) quantification of 5′tRF Gly‐GCC and Glu‐CTC was performed using custom designed TaqMan primers (ID: CTRWEPD; ID: CTWCW97). The validation of this method was reported previously.^20^, ^21^

### 2.4 Small RNA sequencing analysis of human CSF

Small RNA sequencing (RNA‐seq) was performed on CSF samples obtained from TLE patients and matched controls. Total RNA extraction including small RNAs was performed using 350 μL of CSF, for each sample, utilizing the miRNeasy Serum/Plasma Advanced Kit (Qiagen) according to the manufacturer's protocol. QIAseq miRNA Library Kit (Qiagen) was used to carry out library preparation. This kit includes magnetic beads for library size selection, which generates small RNA‐seq libraries with a broad size profile including tRNA‐derived stress‐induced RNAs (tiRNAs). Subsequently, Agilent 2100 Bioanalyzer and the Qubit High Sensitivity DNA Assay Kit from Thermo Fisher Scientific were used to assess the quality and concentration of the resulting libraries. All libraries were pooled in equimolar amounts, and the Illumina NovaSeq X Plus platform was used for sequencing using 150‐cycle single‐end sequencing.

### 2.5 Small RNA‐seq analysis of plasma from TLE patients and healthy controls

Human plasma small RNA‐seq samples were obtained from a previously published study. Analogous to the CSF dataset above, the plasma samples also included TLE patient and healthy control groups. The full description of the patient cohort and methodology for RNA extraction and sequencing can be found in the original study.^12^

### 2.6 tRF analysis

tsRNAsearch,^22^ an established Nextflow pipeline designed to identify and quantify sncRNAs, including tsRNAs and microRNAs, was used to analyze both CSF and plasma small RNA‐seq data. tsRNAsearch comes with the capability to perform differential expression analysis, but to allow for more flexibility and control over plotting and statistical modeling, we used DESeq2^23^ in R programming language to perform our own differential expression analysis. Finally, false discovery rate‐corrected differentially expressed features using the Benjamini–Hochberg method were identified. The primary focus of our small RNA‐seq analysis was to assess the levels of Glu‐CTC and Gly‐GCC 5′ tRNA‐derived fragments.

### 2.7 Statistical analysis

Regarding behavior and EEG analysis of epilepsy in rats, data were analyzed using GraphPad Prism 8.0.2, and paired *t*‐tests (*p* < .05) were employed to compare seizure frequency and duration across different time points. The Grubbs test (alpha = .05) was used to identify and remove outliers. Mean daily frequency of behavioral or EEG seizures was determined by averaging the total number of seizures observed in each animal per day (expressed as SRSs/day ± SEM; see Results section). Mean duration of individual motor or EEG seizures was calculated as follows: (1) average duration of all seizures observed in a single animal on a given day was calculated and (2) mean of daily average durations across all observation days was then determined.

Receiver operator characteristic (ROC) analysis was performed to assess the discriminative ability between control and TLE rat samples, using GraphPad Prism (v10.5). The analysis evaluated the ability of the tsRNAs to correctly classify the TLE rats as the positive condition relative to the control rats as a negative condition. For this analysis, the tRF levels measured in the five plasma and CSF samples taken from each individual control or epileptic rat were averaged, obtaining a single measure per animal. The area under the curve (AUC) was calculated as a measure of overall diagnosis performance, where higher values indicate a better discrimination between TLE rats and control.

Statistical analysis of qPCR data was performed by using Mann–Whitney, two‐way analysis of variance, and post hoc Kruskal–Wallis test. In addition, linear mixed‐effects models were employed to account for repeated measurements within animals, incorporating a rat‐specific random intercept; fixed effects were specified according to the experimental design.

## 3 RESULTS

### 3.1 Spontaneous seizures and IS activity in TLE rats

**FIGURE 1:**
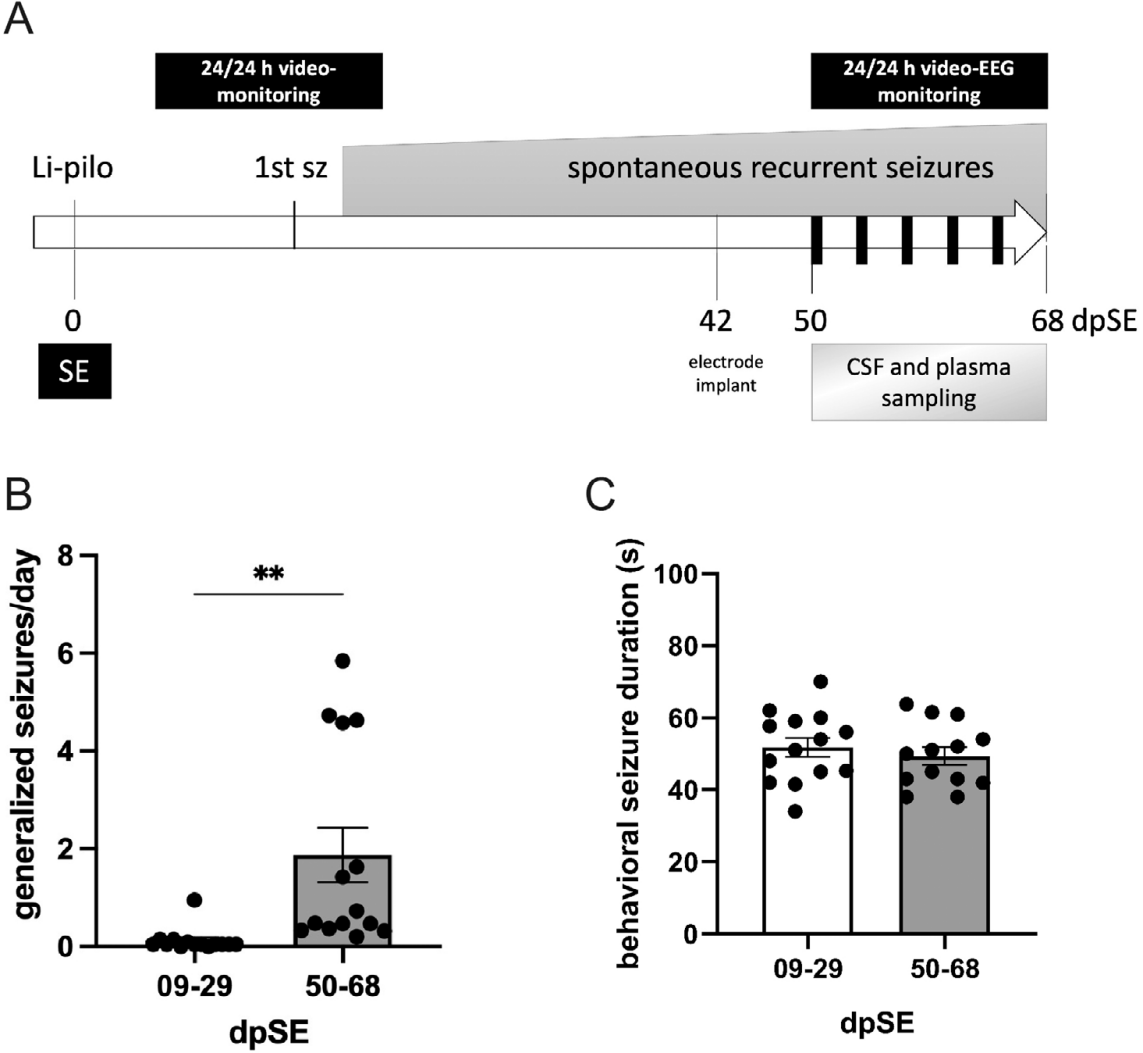
Epilepsy progression in temporal lobe epilepsy rats. (A) Experimental design showing withdrawal time points (thick black bars, 50–65 dpSE; see text for details). (B, C) Average frequency (B) and duration (C) of spontaneous generalized motor seizures (classes 4 and 5) in epileptic rats during the early chronic (9–29 dpSE, white bars) and late chronic (50–68 dpSE, gray bars) periods following SE. The significant increase in seizure frequency in the late chronic phase indicates disease progression. Data are presented as mean ± SEM of *n* = 14 animals per group. **p < .01, paired *t*‐test. CSF, cerebrospinal fluid; dpSE, days post‐SE; EEG, electroencephalographic; Li‐pilo, lithium–pilocarpine; SE, status epilepticus; sz, seizure.

To verify that the animals in the present study exhibited the characteristics of epilepsy development and progression reported in previous studies, we video‐monitored them at an early chronic phase (9–29 days post‐SE) to verify the occurrence of motor SRSs and then video‐EEG‐monitored at a late chronic phase (50–68 days post‐SE), while collecting samples of CSF and plasma (Figure 1A). Pilocarpine (administered after lithium and methyl‐scopolamine pretreatment) induced convulsive SE with onset after 38.2 ± 3.5 min (*n* = 14 rats), which was terminated after 2 h using an antiseizure drug cocktail. For 2–3 days following SE, the animals experienced occasional, self‐limiting generalized seizures (<1 min in duration) and then entered a latency period characterized by apparent well‐being.^17^, ^18^, ^24^, ^25^, ^26^, ^27^ The first spontaneous seizure occurred 21 ± 2 days after SE (mean ± SEM).

In the early chronic phase (1 week after the first SRS), the mean frequency of generalized (class 4 and 5) motor seizures was .1 ± .1 SRSs/day (mean ± SEM; *n* = 14). In the late chronic phase (50–68 days post‐SE), generalized seizure frequency significantly increased, up to 1.9 ± .6 SRSs/day (*p* < .01, paired *t*‐test), indicating disease progression (Figure 1B). The duration of single motor seizures was not significantly different between the early and late chronic periods (early: 52 ± 3 s, late: 49 ± 2 s, mean ± SEM; Figure 1C). Video‐EEG recording of animals in the late period also allowed detection of nonmotor seizures. Specifically, the overall EEG‐based seizure frequency was 2.6 ± .7/day, with a mean duration of 65 ± 2 s. Thus, the duration of spontaneous seizures detected by EEG was, as expected, longer than the motor phase that was observed in a subset (approximately 70%) of cases. Control animals did not display any seizure activity.

During the 50–68 days post‐SE period, rats were also monitored for the presence of ISs in EEG recordings. ISA activity was considered present if its total duration exceeded 480 s within 2‐h windows.

### 3.2 Quantification of 5′tRF Glu‐CTC and Gly‐GCC tRFs in CSF and plasma from TLE rats

**FIGURE 2:**
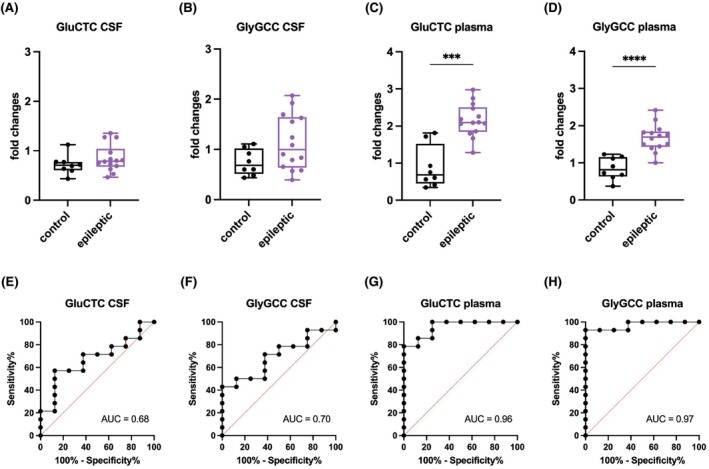
5′ transfer RNA fragment (tRF) Glu‐CTC and 5′tRF Gly‐GCC in rat CSF and plasma. (A–D) Relative levels of Gly‐GCC and Glu‐CTC in CSF (A, B) and plasma (C, D) taken from control (*n* = 8) and epileptic (lithium–pilocarpine‐treated) rats (*n* = 14), determined by real‐time quantitative polymerase chain reaction and normalized to Cel‐miR‐39‐3p and let‐7c‐5p. ***p < .001, ****p < .0001, Mann–Whitney test. (E–H) Receiver operator characteristic analysis of 5′tRF Glu‐CTC and Gly‐GCC levels taken from CSF and plasma of control versus epileptic rats. AUC, area under the curve; CSF, cerebrospinal fluid.

First, we investigated levels of 5′tRF Glu‐CTC and Gly‐GCC in CSF and plasma samples from rats in the lithium–pilocarpine model of TLE, without taking account of the time from the most recent seizure. The relative levels of 5′tRF Glu‐CTC and Gly‐GCC were slightly, but not significantly, different between CSF samples taken from control and epileptic animals (Figure 2A,B). ROC analysis was performed to evaluate the ability of the tsRNAs to discriminate between TLE rats (positive class) and controls (negative class). The AUC of 5′tRF Glu‐CTC and Gly‐GCC of the ROC curve were .68 and .70, respectively (Figure 2E,F), indicating modest discriminative performance.

In contrast, the relative levels of 5′tRF Glu‐CTC and Gly‐GCC in plasma samples taken from epileptic animals were significantly higher than in the control group (Figure 2C,D). A ROC analysis revealed highly specific and sensitive prediction value (AUC of .96 for 5′tRF Glu‐CTC and .97 for Gly‐GCC; Figure 2G,H). Taken together, these data demonstrate that plasma but not CSF levels of 5′tRF Glu‐CTC and Gly‐GCC are elevated in rats with chronic epilepsy. However, one caveat could be that these results were obtained by pooling measures from multiple samples taken at different timepoints. We therefore asked if individual, single plasma samples would consistently provide similarly significant results. We performed separate ROC analyses by comparing individual plasma samples collected at different timepoints in epileptic and control animals. As shown in Figure S1, this analysis found significant differences in most, but not all, cases. In a translational perspective, it would therefore be advisable to perform repeated sampling and measures.

### 3.3 Correlation of ictal epileptiform activity with biofluid levels of 5′tRF Glu‐CTC and Gly‐GCC in rats

**FIGURE 3:**
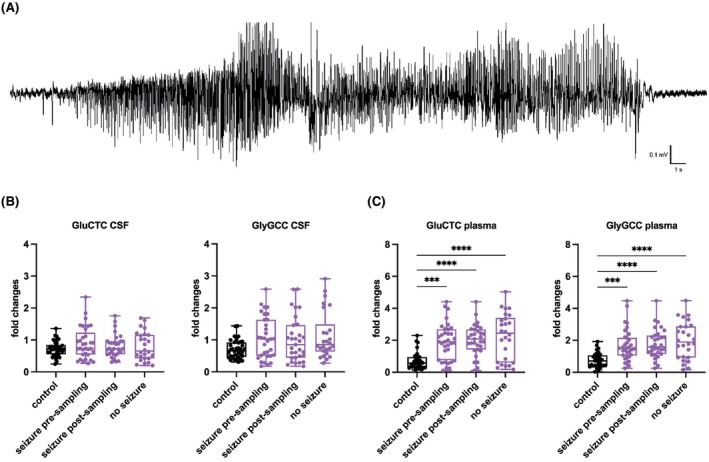
Levels of 5′ transfer RNA fragment (tRF) Glu‐CTC and Gly‐GCC in relation to seizure activity. (A) Representative trace of a typical electroencephalographic seizure. Note that the majority of spikes exhibit amplitudes that exceed three times the peak‐to‐peak noise level. (B) Levels of 5′tRF Glu‐CTC and Gly‐GCC in the CSF. (C) Levels of 5′tRF Glu‐CTC and Gly‐GCC in plasma. Seizure presampling indicates that samples were collected from epileptic rats that experienced seizures in the 24 h prior to sampling. Seizure postsampling means that samples were collected from epileptic rats that experienced seizures in the 24 h following sampling. No seizure indicates that samples were collected from epileptic rats that did not experience seizures 24 h before nor 24 h after sampling. ***p < .001, ****p < .0001, analysis of variance and post hoc Kruskal–Wallis test. CSF, cerebrospinal fluid.

Next, we explored whether the levels of 5′tRF Glu‐CTC and Gly‐GCC varied in relation to recent seizure activity. Therefore, we classified plasma and CSF samples depending on whether a seizure occurred prior to or after the collection of CSF or plasma. Samples were grouped into three categories: (1) animals that experienced one or more seizures (as in Figure 3A) in the 24 h before sampling, (2) animals that experienced one or more seizures in the 24 h after sampling, and (3) animals with no seizures in the 24 h before or 24 h after sampling. Interestingly, 5′tRF Glu‐CTC and Gly‐GCC levels were slightly but not significantly increased in CSF samples from all three subgroups (Figure 3B), whereas they were highly significantly increased in all subgroups in plasma (Figure 3C). Similar findings were observed when seizures occurred within 2 h of sampling (data not shown). These data suggest that the changes in tRF levels (in particular, the increases in plasma 5′tRF Glu‐CTC and Gly‐GCC) are associated with established epilepsy but do not fluctuate significantly as a function of the occurrence of individual spontaneous seizures.

### 3.4 Correlation of IS activity with the levels of 5′tRF Glu‐CTC and Gly‐GCC levels in rat TLE

**FIGURE 4:**
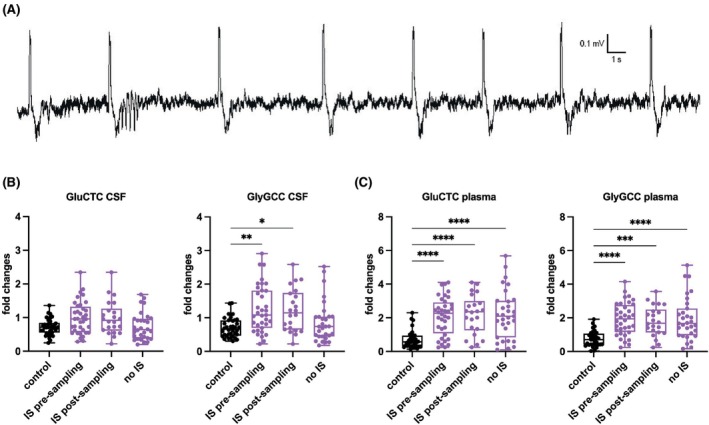
Levels of 5′ transfer RNA fragment (tRF) Glu‐CTC and Gly‐GCC in relation to IS activity. (A) Representative trace of a typical electroencephalogram displaying regular IS activity. (B) Levels of 5′tRF Glu‐CTC and Gly‐GCC in the CSF. (C) Levels of 5′tRF Glu‐CTC and Gly‐GCC in plasma. IS presampling indicates that samples were collected from epileptic rats that experienced IS activity in the 2 h prior to sampling. IS postsampling indicates that samples were collected from epileptic rats that experienced IS activity in the 2 h following sampling. No IS means that samples were collected from epileptic rats that did not display ISs 2 h before or after sampling. *p < .05, **p < .01, ***p < .001, ****p < .0001, analysis of variance and post hoc Kruskal–Wallis test. CSF, cerebrospinal fluid; IS, interictal spike.

ISs are brief, paroxysmal electrographic discharges detected in EEG recordings that occur between SRSs.^4^ Although the pathologic significance and biomarker value of ISs remain incompletely understood, rats with TLE, similar to humans, often display ISs in the lithium–pilocarpine model (Figure 4A). We therefore sought to determine whether levels of 5′tRF Glu‐CTC and Gly‐GCC in CSF and plasma change as a function of IS activity. For this purpose, ISs were defined as the presence of repeated, regular ISs (one every ~5 s) for >8 min within a 2‐h interval. Animals were grouped into whether they experienced IS activity in the 2 h before sampling, in the 2 h after sampling, or showed no IS activity in the 2 h before or after sampling.

Plasma levels of 5′tRF Glu‐CTC and Gly‐GCC were significantly elevated in all subgroups of rats with TLE, and there were no differences in levels according to temporal proximity to IS activity (Figure 4C). In contrast, we observed significantly higher levels of 5′tRF Gly‐GCC in CSF samples taken before and after IS activity, but not in those collected when no ISs were observed either before or after sampling (Figure 4B). Similar results were obtained for CSF levels of 5′tRF Glu‐CTC, displaying higher, albeit not statistically significantly, levels in samples collected before and after IS activity but no elevation in samples collected at times without IS activity (Figure 4B). These data suggest that the increased levels of plasma 5′tRF Glu‐CTC and Gly‐GCC are associated with the condition of epilepsy but do not fluctuate significantly as a function of the occurrence of ISs. In contrast, CSF levels of 5′tRF Gly‐GCC increase specifically in association with IS activity.

Although of limited statistical power, we also conducted mixed‐effects modeling to account for the primary statistical comparisons being based on group‐level visualization and not explicitly modeling within‐animal correlation arising from repeated sampling. The mixed‐effects results (Tables S1 and S2) suggest that between‐animal baseline differences account for a substantial proportion of variability, whereas seizure burden and recency contribute with modest explanatory power in this dataset.

### 3.5 5′tRF levels are higher in plasma than CSF in human TLE

**FIGURE 5:**
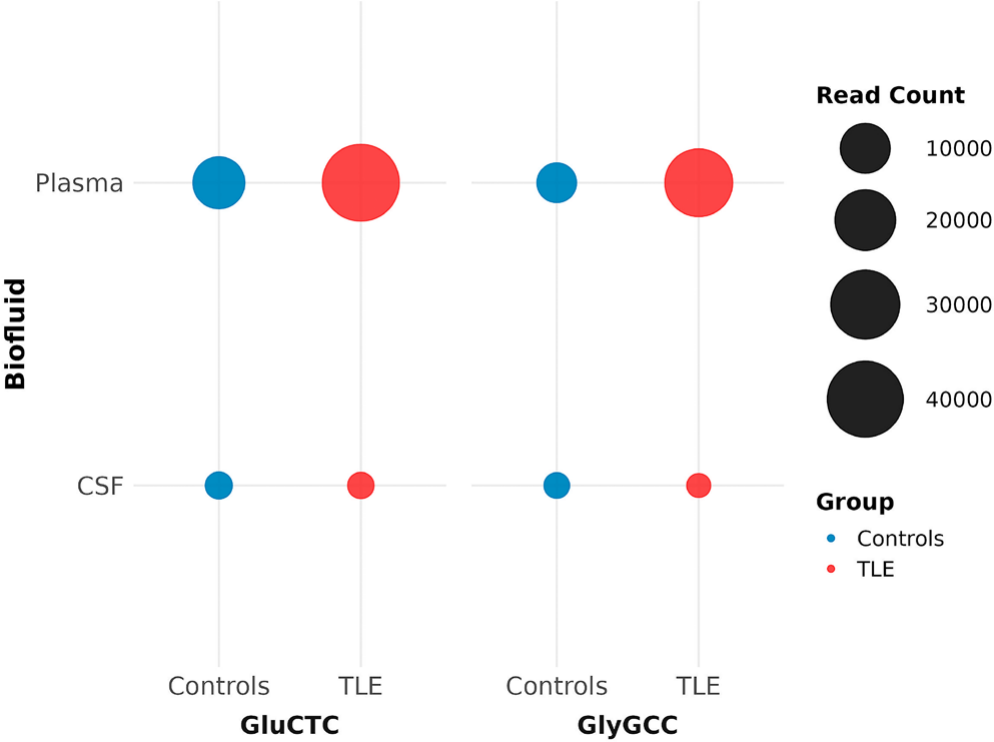
Read counts of 5′ transfer RNA fragment (tRF) Glu‐CTC and Gly‐GCC across biofluids (cerebrospinal fluid [CSF] and plasma) and experimental groups. Read counts (mean across samples per experimental group) of 5′tRF Glu‐CTC and Gly‐GCC are shown in plasma and CSF from two experimental groups (healthy controls and temporal lobe epilepsy [TLE] patients). A very high abundance of both 5′tRFs, with significantly higher abundance in TLE patients compared to healthy controls, is observed in plasma in contrast to CSF, which is marked by lower read content of both tRFs; the control group exhibits similar reads as the TLE group.

To validate our experimental findings in rats with TLE, we analyzed small RNA‐seq data from plasma and CSF samples collected from TLE patients and controls, with a particular focus on the levels of 5′tRF Glu‐CTC and Gly‐GCC. In plasma, we observed increased levels of both 5′tRF Glu‐CTC and Gly‐GCC fragments in TLE patients compared to controls, consistent with the elevated levels observed in our plasma qPCR analysis. In contrast, CSF samples exhibited very low levels of these two fragments, and there were no differences between TLE patients and controls (Figure 5). These small RNA‐seq results align closely with our findings in the rat model, particularly in demonstrating that the elevated levels of 5′tRF Glu‐CTC and Gly‐GCC are primarily detectable in plasma and not in CSF.

## 4 DISCUSSION

The findings of this study demonstrated that levels of 5′tRF Gly‐GCC and Glu‐CTC were significantly elevated in the plasma of rats following lithium–pilocarpine‐induced TLE compared to control rats. The diagnostic performance of the fragments was evaluated using ROC analysis. The analysis determined AUC values of .97 and .96 for the respective 5′tRFs, indicating high discriminative accuracy. In epileptic animals, however, plasma levels did not correlate with the occurrence of individual seizures within 24 h (or 2 h) of sampling. In contrast to plasma levels, there were only moderately higher levels of 5′tRF Gly‐GCC and Glu‐CTC in the CSF of epileptic animals, which, however, did not reach statistical significance. Interestingly, CSF levels of 5′tRF Gly‐GCC were found to increase specifically in association with IS activity. We also attempted to validate aspects of these findings in TLE patients. In line with our previous report,^12^ we demonstrated elevated 5′tRF levels in the plasma of TLE patients when compared to control subjects. However, CSF levels were not elevated in TLE patients.

In recent years, there has been an increase in research interest in potential biofluid‐based diagnostic markers for epilepsy, driven by the need for minimally invasive tools to facilitate diagnosis and monitoring. Among the emerging molecular candidates, sncRNAs have gained noticeable attention for their stability in serum, plasma, and CSF.^28^, ^29^, ^30^ Among the emerging candidates, 5′tRFs have been observed to exhibit altered levels in samples obtained prior a seizure, making them a potential predictor of seizures.^12^ The present study addresses the cross‐species conservation of previous observations in humans by examining its replication in an experimental model of TLE and extends the analysis to CSF. As the plasma had previously been assessed for the presence of 5′tRFs, it is intuitive to consider that CSF would offer a more precise reflection of the molecular alterations associated with epileptiform activity. Due to its proximity to brain tissue and reduced dilution by peripheral factors, CSF was expected to offer a more accurate representation of the processes occurring in the brain.

The most significant finding in the present study was that plasma samples from rats with TLE also displayed elevated levels of 5′tRFs previously associated with TLE. The finding aligns with the small RNA‐seq data of human samples and the previous study.^12^ This suggests that the peripheral biofluids may serve as a reflection of central molecular alterations that occur during chronic epilepsy. Overall, this emphasizes the potential of 5′tRFs as diagnostics for TLE, with plasma‐based detection offering a noninvasive and nonexpansive approach that may integrate the EEG.

Recent work by Vaknine Treidel et al.^31^ showed that specific 5′tRF families are regulated in response to psychosocial stress. Our data are conceptually aligned, showing selective regulation of 5'tRF Glu‐CTC and 5'tRF Gly‐GCC in epilepsy. Unlike with prenatal stress, we show that these changes are compartment‐specific and relate to interictal activity, suggesting that 5'tRF induction may reflect neural hyperexcitability‐associated perturbations rather than generalized stress. Together, these findings extend stress‐responsive tRF regulation to a neurological disease context.

The second interesting finding was that CSF levels of 5′tRF Gly‐GCC were significantly higher in epileptic animals that showed IS activity within 2 h of sampling. This could be evidence of a dynamic regulation of 5′tRF Gly‐GCC levels in animals with acutely hyperactive networks. The routes of 5′tRF secretion during hyperexcitation require further analysis. An attractive hypothesis is the secretion of tRNA fragments via extracellular vesicles, as observed in other neurological diseases where the release of sncRNAs has been reported.^32^, ^33^, ^34^ Monitoring 5′tRF levels in CSF, although more invasive, could be a promising approach for patient stratification in a clinical setting.

However, CSF levels of tRF Gly‐GCC correlated with proximity with ISs but not with proximity to seizures. How can this apparent discrepancy be explained? One possibility is that ISs reflect sustained network hyperexcitability and cellular stress responses that promote local tRF generation or release within the central nervous system (CNS), whereas seizures may trigger rapid redistribution or systemic clearance mechanisms. Seizures are known to transiently disrupt the blood–brain barrier (BBB), allowing bidirectional exchange between blood and CSF that could result in a flooding of 5′tRF levels into the blood.^35^ In this context, transient BBB opening could contribute to a shift of CNS‐derived 5′tRFs into the circulation, thereby attenuating detectable CSF changes while contributing to persistently elevated plasma levels. However, alternative explanations must also be considered. tRNA fragments are not brain‐specific molecules and are generated in multiple peripheral tissues under conditions of cellular stress and inflammation.^36^ Thus, elevated plasma levels may reflect systemic responses associated with chronic epilepsy rather than exclusively CNS‐derived release. The association of 5′tRF Gly‐GCC with IS activity in CSF nevertheless supports at least a partial link to central network activity.

tRFs may also associate with Ago proteins^37^ or extracellular vesicles that can stabilize them in biological fluids, preventing their degradation by nucleases and influencing their compartmental distribution. 5′tRF levels in plasma may therefore memorize seizures. However, we can presently not exclude that plasma levels of 5′tRFs originate from sites other than the CNS in TLE patients. Although a memorizing effect is advantageous in terms of their diagnostic potential for TLE, plasma‐based detection of 5′tRFs may be less suitable for dynamic monitoring of seizure imminence. Nevertheless, because the BBB is permeable for structures under 10 kDa,^38^ we cannot exclude the possibility that tRFs are produced outside the CNS and enter the CNS in an EEG‐active interictal period. Importantly, our data do not allow definitive determination of tissue origin. Future studies combining brain tissue profiling, cell type‐specific analyses, and paired CSF–plasma extracellular vesicle characterization will be required to resolve the relative contributions of central versus peripheral sources.

A limitation of the present analysis is that the primary statistical comparisons were based on group‐level visualization. Although mixed‐effects modeling was performed to account for within‐animal correlations, the relatively small number of animals limited statistical power to detect moderate seizure‐related effects. Our statistical analyses may therefore be interpreted with a degree of caution, and validation in larger longitudinal cohorts is warranted.

## 5 CONCLUSIONS

Our findings provide a biological validation for the use of 5′tRFs as potential biofluid biomarkers in TLE. The presence of 5′tRFs in the plasma has proven to be a reliable indicator of an underlying epileptic state, although they cannot discriminate the proximity to recent seizures or IS activity. However, we found that the levels of 5′tRF Gly‐GCC in CSF increase specifically in association with IS activity. The accessibility in biofluids and their ease of detection make tRFs good candidates as potential biomarkers for diagnosis and for a cost‐effective patient monitoring.

## AUTHOR CONTRIBUTIONS

Michele Simonato and Jochen H. M. Prehn were involved in conception and design of the study. Marie Soukupova, Elena Perez Morrissey, Ina Woods, Annunziata Guarino, Pietro Marino, Cristiana Pareo, and Nicolò Birtolo carried out research and data acquisition. Saad Zaheer carried out bioinformatics analyses. Elena Perez Morrissey, Marie Soukupova, Saad Zaheer, Rachel Stewart, Michele Simonato, and Jochen H. M. Prehn carried out analysis and interpretated data. Hajo Hamer and Péter Körtvélyessy collected human samples and clinical data. Michele Simonato, Jochen H. M. Prehn, Elena Perez Morrissey, Marie Soukupova, Shona Pfeiffer, and David C. Henshall wrote the manuscript. Felix Rosenow, Hajo Hamer and Péter Körtvélyessy collected human samples and clinical data. All authors reviewed and approved the manuscript prior to submission.

## FUNDING INFORMATION

M.Si., J.H.M.P., and D.C.H. were supported by the European Union FET project PRIME—A Personalised Living Cell Synthetic Computing Circuit for Sensing and Treating Neurodegenerative Disorders (H2020 FET–GA 964712). J.H.M.P. and D.C.H. were also supported by Taighde Éireann–Research Ireland under grant number 21/RC/10294_P2 at FutureNeuro Research Ireland Centre for Translational Brain Science. S.Z. was supported by Taighde Éireann–Research Ireland Centre for Research Training in Genomics Data Science under grant number 18/CRT/6214 and the European Union Seventh Framework Programme under grant agreement no. 602130 (EpimiRNA).

## CONFLICT OF INTEREST STATEMENT

None of the authors has any conflict of interest to disclose.

## ETHICS STATEMENT

All animal experiments were approved by the University of Ferrara institutional animal care and use committee and by the Italian Ministry of Health (approval no. CBCC 2.52 [603/2022‐PR]) and were performed in accordance with the guidelines of the European Community Council Directives 2010/63/EU as well as the ARRIVE and the NC3Rs (National Center for the Replacement, Refinement, and Reduction of Animal Research) guidelines. Written informed consent was obtained from all participants prior to their enrollment in the study. The human study was conducted in accordance with the Declaration of Helsinki, and the protocol was approved by the institutional review board/ethics committee of Magdeburg (approval no. 07/17) and Erlangen (approval no. 128/14Bc). We confirm that we have read the Journal's position on issues involved in ethical publication and affirm that this report is consistent with those guidelines.

## Supporting information


**FIGURE S1** Receiver operator characteristic analysis of Glu‐CTC (top panels) and Gly‐GCC 5′ transfer RNA fragment (bottom panels) levels taken from plasma of control versus epileptic rats at each of the five sampling times (see Materials and Methods for details on the sampling).

**TABLE S1** Linear mixed‐effects model results for cerebrospinal fluid 5′ transfer RNA fragment fold change.
**TABLE S2** Linear mixed‐effects model results for plasma 5′ transfer RNA fragment fold change.

## Data Availability

The data that support the findings of this study are available on request from the corresponding author.
