# Supplementary figures and images for: Biofluid‐specific variations in circulating 5′ transfer RNA fragments during ictal and interictal states in experimental temporal lobe epilepsy
Source: Epilepsia. 2026 Apr 13;67(7):3803–14. doi: 10.1002/epi.70246 (PMC13360991; doi:10.1002/epi.70246)

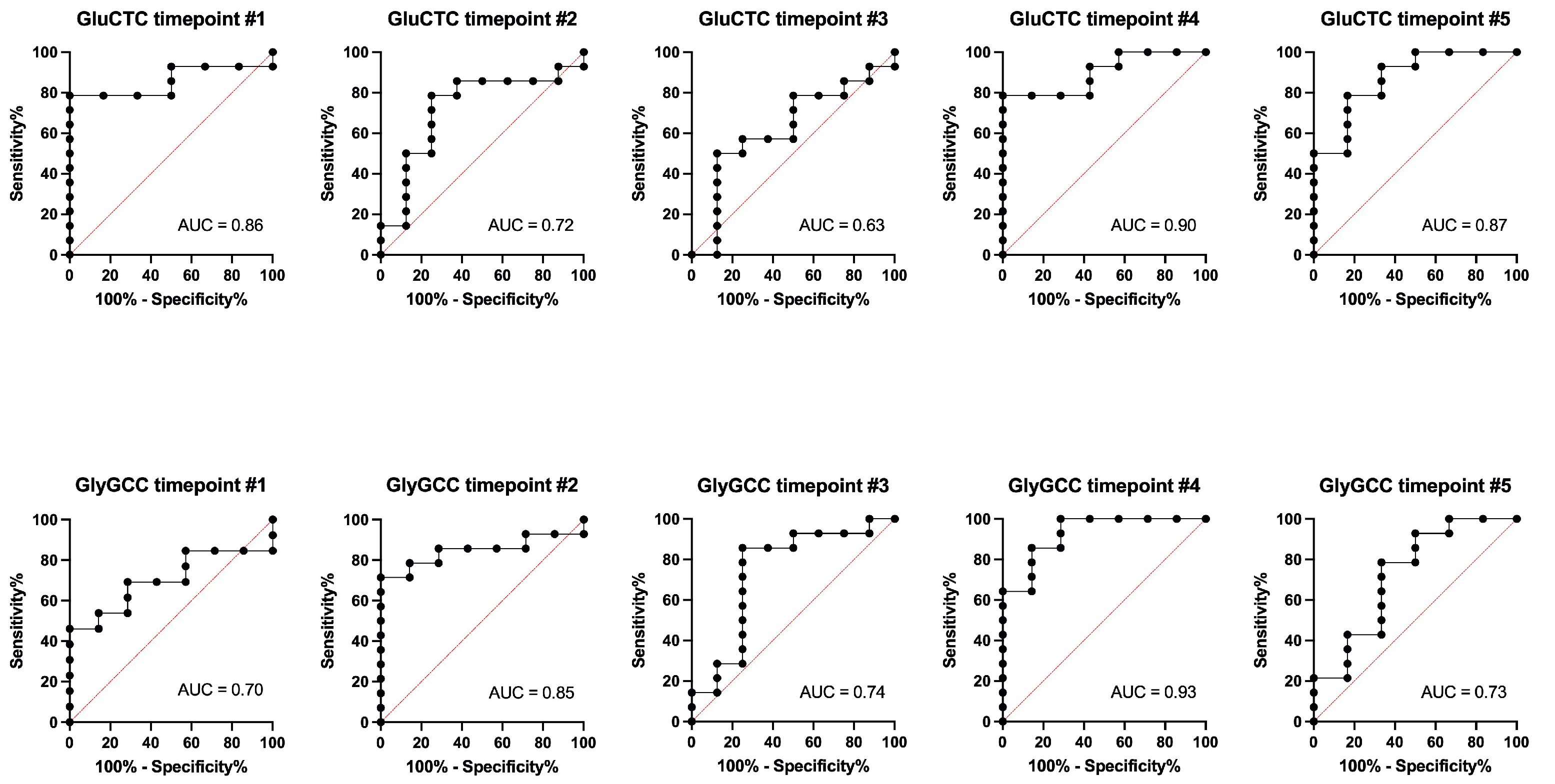

Supplement: Supplementary file 1 — FIGURE S1 Receiver operator characteristic analysis of Glu‐CTC (top panels) and Gly‐GCC 5′ transfer RNA fragment (bottom panels) levels taken from plasma of control versus epileptic rats at each of the five sampling times (see Materials and Methods for details on the sampling). [file EPI-67-3803-s002.tif]
