# Supplementary material for: Biofluid‐specific variations in circulating 5′ transfer RNA fragments during ictal and interictal states in experimental temporal lobe epilepsy
Source: Epilepsia. 2026 Apr 13;67(7):3803–14. doi: 10.1002/epi.70246 (PMC13360991; doi:10.1002/epi.70246)
Supplement: Supplementary file 2 — TABLE S1 Linear mixed‐effects model results for cerebrospinal fluid 5′ transfer RNA fragment fold change. TABLE S2 Linear mixed‐effects model results for plasma 5′ transfer RNA fragment fold change. [file EPI-67-3803-s001.docx]

**Biofluid-specific variations in circulating 5’ tRNA fragments during ictal and inter-ictal states in experimental temporal lobe epilepsy**

Marie Soukupova, Elena Perez Morrissey, Annunziata Guarino, Pietro Marino, Cristiana Pareo, Nicolò Birtolo, Saad Zaheer, Rachel Stewart, Ina Woods, Felix Rosenow, Hajo Hamer, Péter Körtvélyessy, Shona Pfeiffer, David C. Henshall, Jochen H.M. Prehn, Michele Simonato

**Supplementary Information**

**Materials and Methods**

**Animals**

Adult male Sprague-Dawley rats (250-350 g; Inotivco, Milan, Italy) were housed under controlled illumination (12 h light/dark cycle; light on 06.00 am) and environmental conditions (ambient temperature 22-24^o^C, humidity 55-65%). Rat chow and tap water were available ad libitum. All efforts were made to reduce animal numbers and suffering during the experiments. All animal studies were performed in accordance with the guidelines of the European Community Council Directives 2010/63/EU as well as the ARRIVE and the NC3Rs (National Centre for the Replacement, Refinement and Reduction of Animal Research) guidelines (1).

**Lithium-pilocarpine model**

All procedures started one week after the arrival of the rats in the animal facility. Animals were then administered 127 mg/kg lithium chloride by gastric gavage and, 14 h later, a subcutaneous injection of methyl-scopolamine (1 mg/kg, Sigma-Aldrich, Saint Louis, MO, USA), a peripherally acting muscarinic antagonist, to mitigate the peripheral adverse effects of pilocarpine. Status epilepticus (SE) was induced 30 min thereafter by administration of pilocarpine (37 mg/kg i.p., Sigma-Aldrich). Motor seizure severity was assessed using the Racine scale (2): stage 1, immobility, eyes closed, and facial clonus; stage 2, head nodding and more pronounced facial twitching; stage 3, clonus of one forelimb; stage 4, rearing with bilateral forelimb clonus; and stage 5, generalized tonic–clonic seizures with rearing and falling. Within 30 min after pilocarpine injection, animals develop continuous, long-lasting generalized seizure activity (stage 4-5), i.e., convulsive SE. If an animal failed to enter convulsive SE within 30 minutes of the initial pilocarpine administration, a second dose of pilocarpine (half the original amount, 18 mg/kg) was administered. Animals that did not develop SE within 30 min after the last pilocarpine administration were excluded from the study. SE was interrupted 2 h after onset by i.p. administration of a cocktail of drugs: diazepam (10 mg/kg), phenobarbital (25 mg/kg), and scopolamine (1 mg/kg). This cocktail was administered again after 4 h. Finally, after another 4 h, rats received an i.p. administration of diazepam and scopolamine only. To aid in recovery from the weight loss that occurs after SE, animals were administered saline injections (1 ml of 0.9% NaCl solution, pH adjusted to 7.4) and provided with a soft palatable food for 2-3 days. Animals that did not regain their pre-SE body weight within a week were excluded from the study. Age-matched control animals received a subcutaneous injection of methyl-scopolamine but were not administered lithium-pilocarpine. In the subsequent 2-3 weeks, animals began to experience spontaneous recurrent seizures (SRSs).

**Surgical implantation of electrodes**

Surgery was performed to implant tethered electrodes or telemetry implants for EEG recordings at 42 or 43 days post-SE, according to previously published procedures (3). Of the 22 rats included in the study, 7 epileptic and 4 control rats were implanted with tethered electrodes, while 7 epileptic and 4 control animals were implanted with telemetry devices. Briefly, rats under ketamine/xylazine (44 and 7.5 mg/kg i.p.) induction anesthesia were placed on a heating pad and secured to a stereotactic apparatus with the nose bar positioned at -3.3 mm. Anesthesia was then maintained using isoflurane (2% in air; 1.2 ml/min). In this study, tethered electrodes (1) and rat EEG telemetry implants (2) were located differently: (1) A tethered bipolar electrode MS333/3-BIU/SPC (Plastics One, Roanoke, VA, USA) was implanted into the right dorsal hippocampus 3.8 mm posterior and -2.5 mm lateral from bregma, 2.7 mm ventral to the surface of the brain. Contralaterally, a stainless-steel screw electrode was implanted on the skull over the right motor cortex to serve as a reference. Three stainless steel screws were inserted into the skull (above the left motor cortex, right somatosensory cortex, and right visual cortex) and served as anchors for dental acrylic. (2) Telemeter electrodes CTA-F40 (Data Sciences International, St Paul, MN, USA) were placed just under the dura, on the surface of the cortex. A recording electrode was placed anterior to bregma over the right hemisphere (2.0 mm anterior and -1.5 mm lateral from bregma), while a reference electrode was placed between bregma and lambda (-4.0 mm posterior and -2.5 mm lateral from bregma). The transmitter was placed into an approximately 2-3 cm wide subcutaneous pocket in the animal's back and sutured to the surrounding tissue. After surgery, all animals received an antibiotic (local 1% micronized silver sulfadiazine) to avoid possible infections and an analgesic drug (tramadol, 7 mg/kg s.c. daily) for 3 days. Rats were allowed to recover for 7 days after surgical procedures while being monitored twice daily for signs of pain or distress.

**Video and video-EEG monitoring**

Animals were continuously (24 h per day/7 days per week) video-recorded for 3 weeks, starting at 9 days post-SE, to score frequency and severity of generalized (stage 4 or 5) SRSs. To this aim, we used a video surveillance system, VIDEOSTAR (Top Italia SRL, Italy). Subsequently, they were implanted with electrodes (as described above) and then underwent video-EEG monitoring between 50 and 68 days post-SE. The tethered system consisted of an amplifier (MP150 Data Acquisition system, Biopac Systems, Goleta, CA, USA) and was paired with video cameras to record animal behavior. EEG signals were amplified 5000 times, band-pass filtered at 0.005 Hz, and then digitized at 0.5 kHz with 12-bit resolution using the data acquisition software AcqKnowledge 5.0 (Biopac, Goleta, CA, USA). The telemetry system consisted of receiver RPC-1, Ponemah post system 6.51, 8-channel media recorder and Ponemah 6 digital cameras (all Data Sciences International), in which signal receivers were assigned to each implanted transmitter. The EEG sampling rate of the system was set at 1000 Hz, noise detection between -500 mV and +500 mV, and the integration interval as 100 ms. NeuroScore 3.4x core software (Data Sciences International) was used for EEG analysis.

**Analysis of epileptiform activity**

All video recordings were visually screened by two independent operators for the detection of motor seizures. Seizure severity was assessed using the Racine scale (2). In cases when the operators disagreed on the presence, severity, or duration of a seizure, they consulted a third reviewer to reach a consensus.

All EEG recordings were visually screened by trained observers, followed by confirmation using the above-mentioned EEG software. An EEG seizure was defined as a high-amplitude (at least three times higher amplitude than baseline), high frequency (>5 Hz) rhythmic discharge lasting longer than 10 sec.

IS activity was defined as regular, repetitive spike-and-wave activity, defined as sharp signals (spike) with an amplitude at least three times greater than the mean baseline activity followed by a slow wave. IS activity was quantified in the 2 h interval preceding or following each CSF and plasma withdrawal. IS activity was considered "present" if detected for at least 480 s (8 min) within the 2 h before or after CSF/plasma withdrawal.

**Within-animal correlation in repeated sampling**

Linear mixed-effects models including a rat-specific random intercept were fitted to account for repeated sampling. Fixed effects included sampling timepoint, log-transformed seizure burden, and recent seizure status (i.e., one or more seizure 24h before or after sampling). Outcomes were log-transformed fold-change values. Marginal R² represents variance explained by fixed effects; Conditional R² represents variance explained by the full model.

Specifically, for each 5’ tRF outcome (GluCTC and GlyGCC), we fit models of the form:

$$y_{ij}= \beta_{0}+\beta_{time}.Timepoint_{j}+\beta_{burden}.\log\left( Burden_{ij}+1 \right)+\beta_{recent}.Recent_{ij}+u_{i}+\epsilon_{ij}$$

where $u_{i}$ represents a rat-specific baseline shift (random intercept) and $\epsilon_{ij}$ is the residual error for each measurement. Fold-change values were log-transformed to stabilize variance and reduce skew.

**Supplementary Table S1.**

**Linear mixed-effects model results for CSF 5’tRF fold-change**


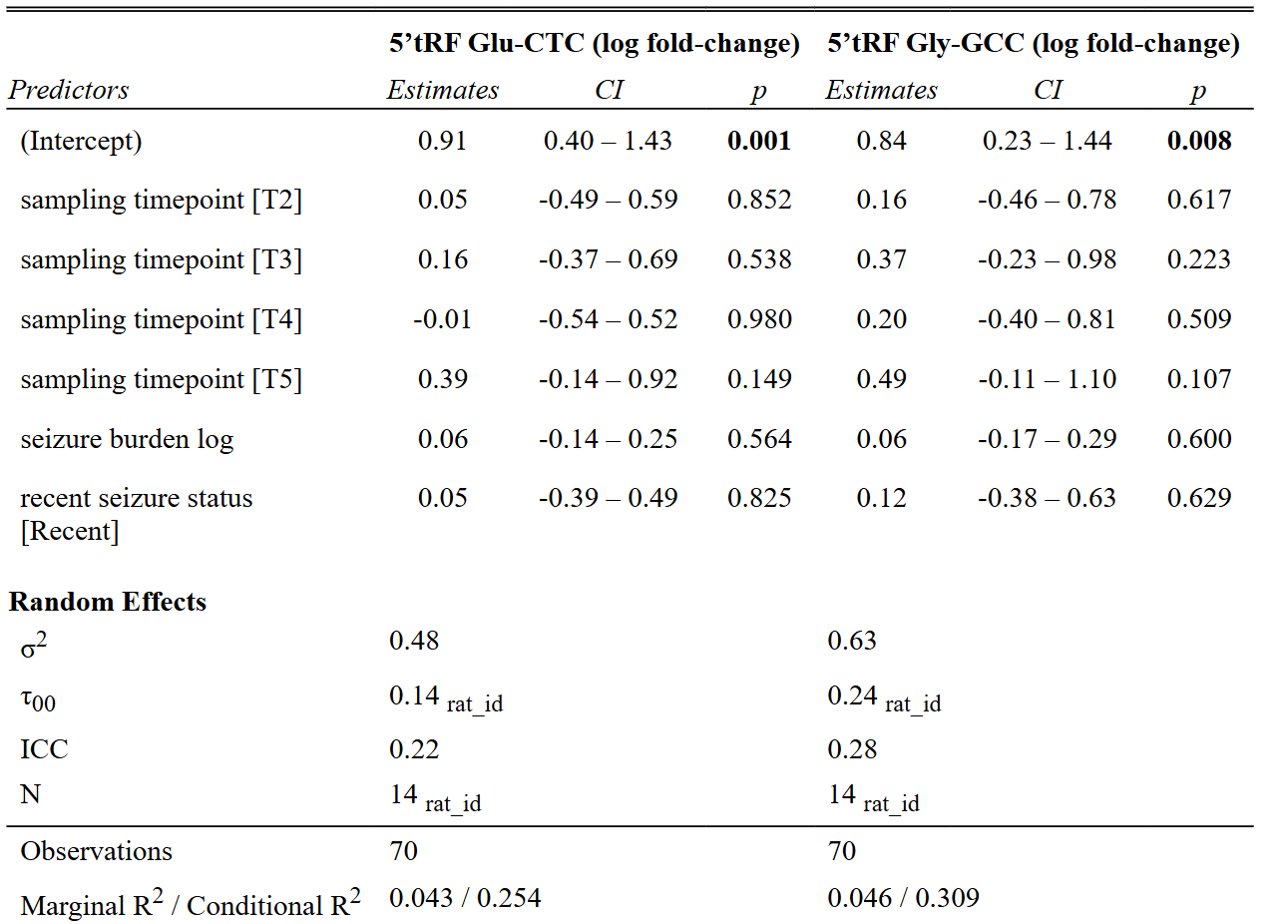


**Supplementary Table S2.**

**Linear mixed-effects model results for Plasma 5’tRF fold-change**

**
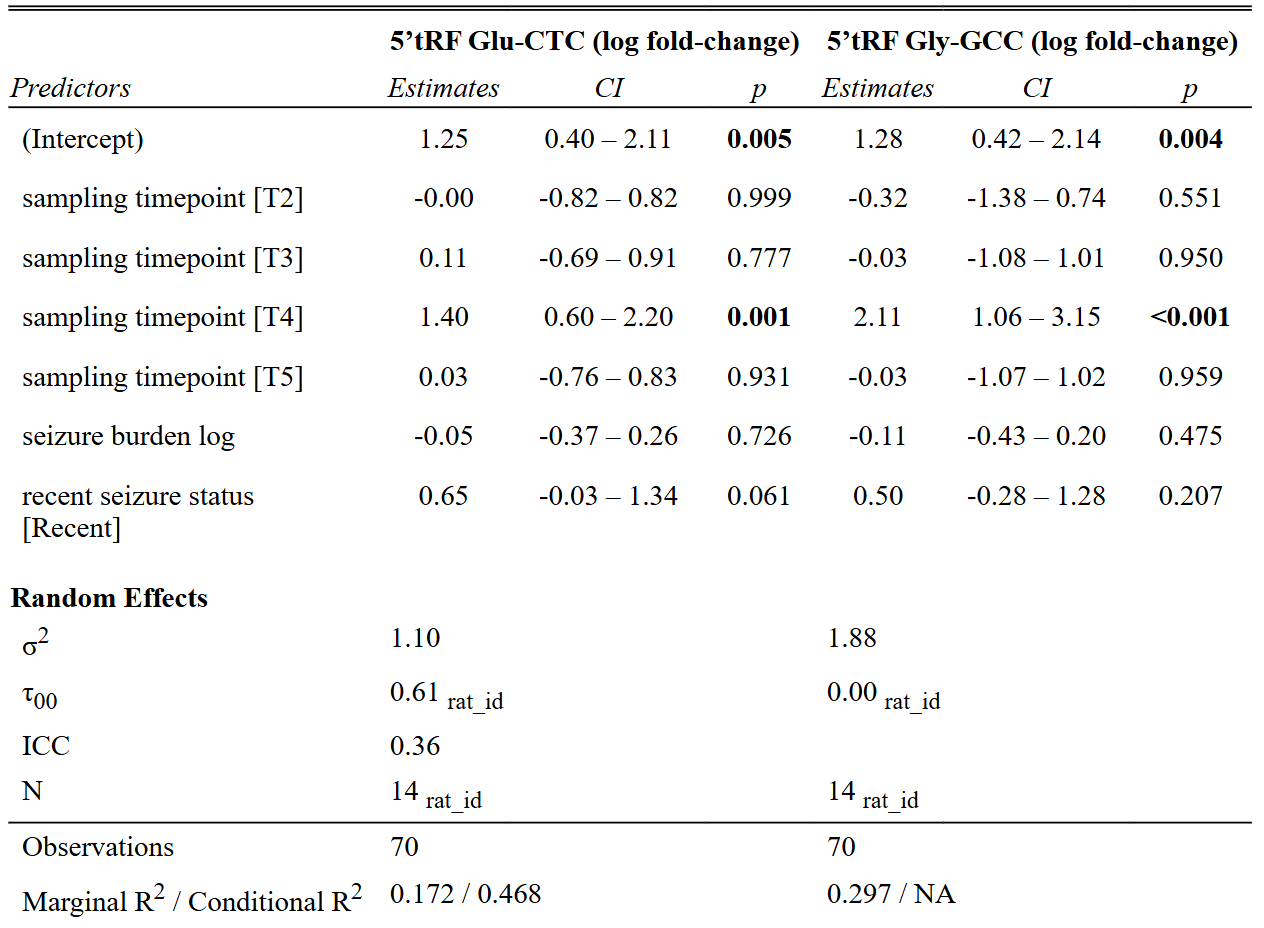
**

**References**

1. Lidster K, Jefferys JG, Blümcke I, Crunelli V, Flecknell P, Frenguelli BG, et al. Opportunities for improving animal welfare in rodent models of epilepsy and seizures. J Neurosci Methods. 2016 Feb 15;260:2–25.

2. Racine RJ. Modification of seizure activity by electrical stimulation. II. Motor seizure. Electroencephalogr Clin Neurophysiol. 1972 Mar;32(3):281–94.

3. Soukupová M, Guarino A, Asth L, Marino P, Barbieri M, Simonato M, et al. Sampling Cerebrospinal Fluid and Blood from Lateral Tail Vein in Rats during EEG Recordings. J Vis Exp. 2023 Sep 1;(199).
